# Case Report of a Rare Clinical Presentation of Craniomaxillofacial Osteosarcoma in an Adult Male Dog

**DOI:** 10.3390/vetsci13080725

**Published:** 2026-07-23

**Authors:** Kirsten E. Rico, Emily Brinker, Mary Labato, Natalie Arruda Bergamaschi, Leslie Schwarz, Celina Morimoto

**Affiliations:** Cummings School of Veterinary Medicine, Tufts University, North Grafton, MA 01536, USAmary.labato@tufts.edu (M.L.);

**Keywords:** oncology, osteosarcoma, bisphosphonate, CT

## Abstract

An 8-year-old intact male German Shorthaired Pointer presented as a referral for a one-week history of unilateral ocular discharge of the left eye and progressive temporalis muscle atrophy. Severe temporalis muscle wasting was appreciated, with periorbital asymmetry. Computed tomography showed aggressive and extensive multifocal bone lysis, primarily on the left rostral aspect of the skull, affecting the cribriform plate, frontal bone, ethmoid bone, zygomatic bone, and maxilla, with marked left temporal muscle atrophy and near-complete collapse and destruction of the left frontal sinus. Bone biopsy revealed osteoblastic osteosarcoma of the skull. Aside from the physical examination abnormalities, the patient did not exhibit pain, lethargy or anorexia at home or while in hospital. This case is notable for the unusual presentation, clinical manifestation and location of osteoblastic osteosarcoma in dogs. The survival time of 132 days was calculated starting from the date of confirmed histopathologic diagnosis, or 159 days from the date of the computed tomography scan.

## Simple Summary

Axial osteosarcomas typically present with radiological evidence of concurrent bone proliferation and lysis. This case report highlights a unique presentation of a dog with an osteoblastic axial osteosarcoma that was primarily lytic on computed tomography imaging and only treated with zoledronate intravenously.

## 1. Introduction

Osteosarcoma (OSA) is a malignant bone tumor and is the most common primary bone tumor in dogs (85%) [1]. There is no cure for this disease, but treatments for appendicular OSA include analgesia, surgery, radiation therapy and chemotherapy [1]. A growing body of evidence in dogs supports breed-associated inheritance of appendicular OSA, especially in Scottish Deerhounds, Rottweilers, Greyhounds, Great Danes, Saint Bernards, and Irish Wolfhounds [1]. The age at diagnosis is typically 7–10 years old, with the highest frequency affecting the metaphyses of long bones of the appendicular skeleton, termed “appendicular OSA”. While appendicular OSA is more common, it can also occur in other regions, including the axial skeleton and extraskeletal sites. The anatomical distribution of skeletal OSA is as follows: the appendicular skeleton comprises about 75% of cases, and the axial skeleton, including ribs, skull, vertebrae and pelvis, comprises about 25% [1]. Axial OSA tends to be more difficult to treat with surgery when compared to appendicular OSA due to the challenge of achieving complete margins.

In appendicular OSA, metastasis is very common and arises early in the course of the disease, although usually subclinically. More than 90% of dogs with appendicular OSA are speculated to have micrometastatic disease because dogs that had no lung metastasis detected on thoracic radiographs prior to receiving amputation surgery have been reported to develop pulmonary metastases within less than one year [2]. The metastatic potential is lower in axial OSA than in appendicular OSA. The reported metastatic rate of axial OSA is variable, ranging from 11 to 58% among retrospective studies [3,4]. One study evaluated 50 dogs with mandibular OSA treated by mandibulectomy. The median survival time was 525 days (17.5 months), and 58% of dogs had metastases at a median of 627 days (20.9 months) [4]. Adjuvant chemotherapy was prognostic for metastasis-free interval and median survival time in multivariate analysis [4]. In a different retrospective report, the median survival for dogs with axial skeletal OSA that received radiation therapy was 137 days (4.6 months), and metastasis was documented in 46% [5]. In that same study, the primary cause of death was local tumor recurrence in 54% of dogs. Another report in 116 dogs with axial OSA treated primarily with surgery showed that the median survival time was 154 days (5.1 months) [3]. In these 116 dogs, about 80% of dogs had the local disease reported as the main cause of death, while pulmonary metastases were detected in only 11% (6 of 54) of dogs who had thoracic radiographs performed. Another study reported the outcome of 183 dogs with OSA of the maxilla, mandible, or calvarium, at which most dogs (74%) received surgery alone. The overall median survival time was 329 days (11 months), and local progression of the disease was reported in 51% of dogs, and a 38% metastatic rate [6]. Hemimaxillectomy in six dogs with osteosarcoma was reported to result in a median survival of 4.6 months, while most of the dogs (5 of 6) died from local or metastatic disease progression [7]. Partial mandibulectomy for 20 dogs with osteosarcoma resulted in a median survival time of 13.6 months, and 9 of 20 dogs died from local or metastatic disease progression [8]. In a different report, partial mandibulectomy alone or in combination with radiation and/or chemotherapy for 49 of 51 dogs resulted in a median survival time of 17.6 months, with a 28% metastatic rate reported [9]. These retrospective studies illustrate that local disease progression is the main concern for dogs with craniomaxillofacial OSA instead of metastatic disease.

Although reports describe the incidence of this subset of OSA individually, it is rarely reported that OSA presents as primarily lytic. The typical behavior of axial OSA is that there is some degree of concurrent proliferation and lysis. This bony proliferation is histologically evident as periosteal new bone formation and, potentially, mineralization of neoplastic osteoid. The unusual aspect of this case is that the computed tomography (CT) scan revealed predominantly lytic bone, consistent with the biopsy’s variable and multifocal osteoid matrix production, which was diagnosed as a variably productive osteoblastic OSA with minimal periosteal new bone formation.

This report will outline the clinical presentation, diagnostic findings, treatments, and outcome of this unique case.

## 2. Case Description

**Figure 1:**
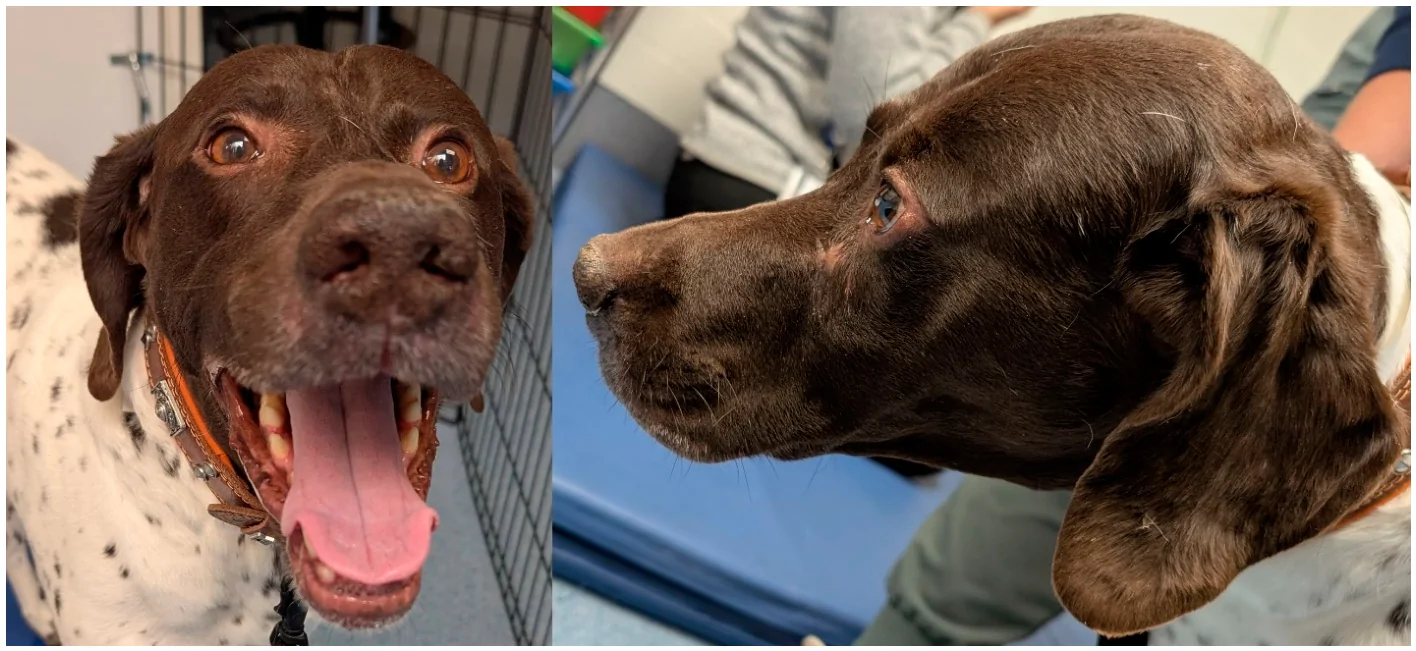
Photos that show the sunken appearance of this dog’s left periorbital region.

An 8-year-old, 27.4 kg (60.28 lbs), male intact German Shorthaired Pointer presented to an emergency and referral hospital for a 1-week history of left ocular discharge and progressive muscle atrophy of the temporalis muscle (Figure 1). The patient had been acting normally at home, including eating normally, having normal energy levels, urinating and defecating normally and not sneezing. The dog presented to the primary care veterinarian 28 days prior for an annual appointment, where the owner had noted an intermittent dry cough of about 4-month duration. The owner noted no other concerns at that time. On physical examination by the primary care veterinarian, no abnormalities were appreciated. Thoracic radiographs were recommended for the cough and were consistent with a bronchial pattern suggestive of chronic lower airway disease. The patient was prescribed doxycycline at 0.35 mg/kg orally twice daily for 10 days and a tapering course of prednisone at 0.35 mg/kg orally twice daily for 3 days, then once daily for 3 days, then every other day for 3 days. Seven days later, the dog was dropped off for sedated thoracic radiographs. The patient was habitually on fluoxetine at 1.4 mg/kg by mouth once daily for anxiety.

Three weeks after the thoracic radiographs were performed, the patient returned to the primary care clinic for epiphora in the left eye. A firm swelling rostral to the medial canthus of the left eye and left-sided temporal muscle atrophy were noted. The patient did not seem bothered by the left eye and appeared otherwise well. The right eye remained unchanged. Fluorescein uptake was negative, and the Schirmer tear test was normal. The primary care veterinarian prescribed clindamycin at 21 mg/kg orally twice daily for 7 days for a potential infection and recommended referral to a specialty hospital for advanced imaging.

On referral presentation, the patient was bright and alert but anxious. The patient was hyperthermic at 40 °C (104 °F), but all other parameters were within normal limits. On physical examination, there was a mild amount of left-sided serous ocular discharge, severe muscle wasting with no palpable underlying bone dorsal to the left eye, causing periorbital asymmetry. No pain was elicited on physical examination, and no other abnormalities were appreciated. An ophthalmology consultation revealed no abnormalities. A complete blood count, a serum chemistry panel, and Brucella testing were performed. The complete blood count revealed no abnormalities; the serum chemistry panel showed an elevated alkaline phosphatase (ALP) at 134 U/L (reference range of 12–127) and an elevated anion gap at 21.1 (reference range 8–19).

**Figure 2:**
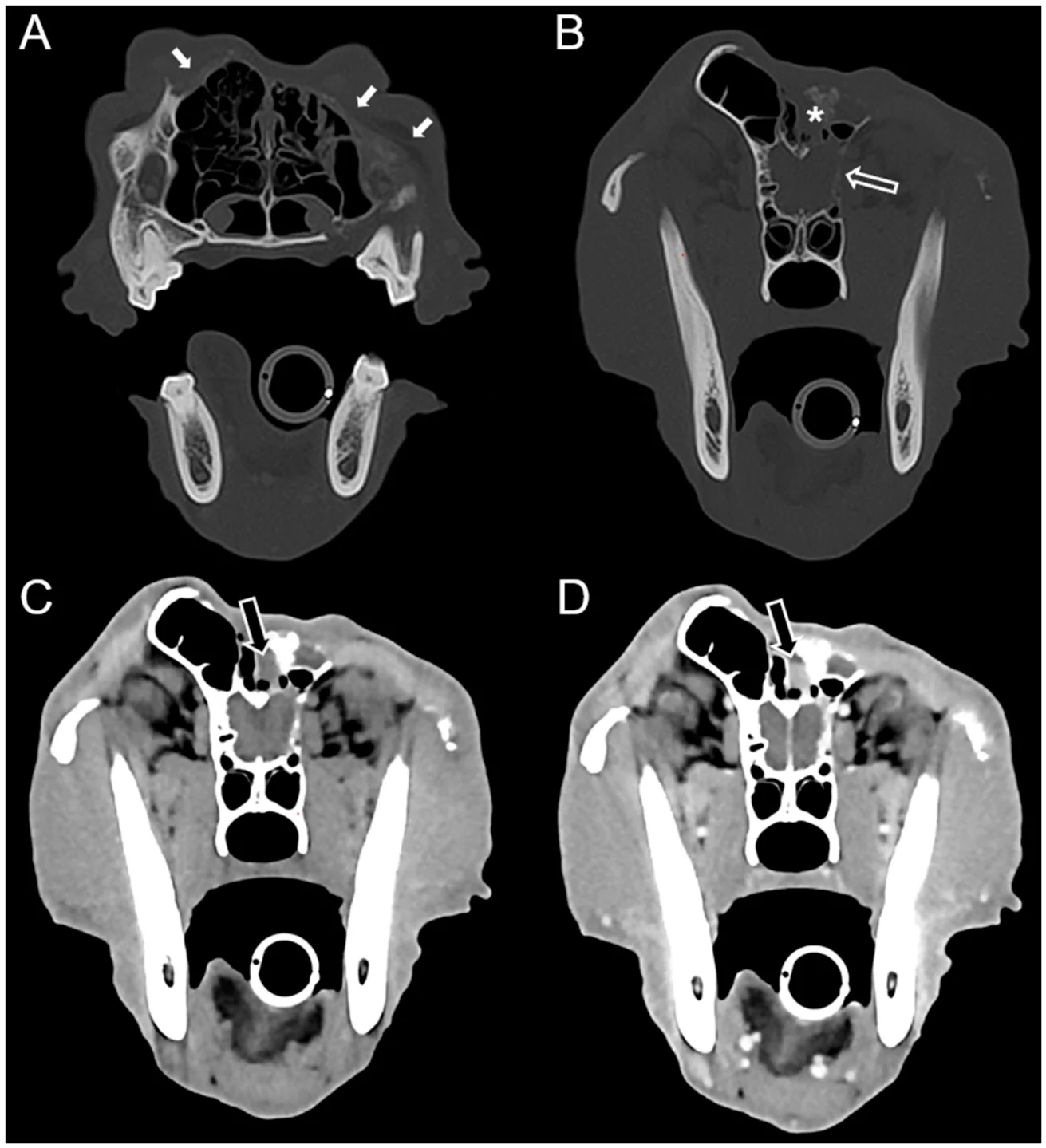
Transverse CT images of the head at the level of the first molar (**A**) and ethmoid turbinates (**B**–**D**). (**A**): Extensive lysis of the nasal bone and maxilla (solid white arrows). (**B**) Communication between the cranial vault and the left retrobulbar space is noted, secondary to multifocal lysis of the orbital part of the frontal bone and the cribriform plate (hollow arrow). Note the collapse of the left frontal sinus (asterisk). (**C**) Pre- and (**D**) Post-contrast images reveal a small region of contrast-enhancing material within the left frontal sinus (black arrows).

A CT scan of the head and thorax with contrast (Omnipaque™, 300 mg/mL; GE Healthcare Inc., Cork, Ireland) was performed on a Toshiba (Tochigi, Japan) multidetector (Canon Aquillon Qalibra, 164-slice) scanner. CT revealed extensive, multifocal lysis, primarily of the left aspect of the skull, and including the nasal, frontal, ethmoid, and zygomatic bones, the maxilla, and cribriform plate. Near complete collapse/destruction of the left frontal sinus and marked left temporal muscle atrophy were also observed (Figure 2). The left medial retropharyngeal lymph node was mildly enlarged. Both infectious and neoplastic processes were considered based on imaging findings. No pulmonary metastasis was appreciated.

Fine needle aspirate of a swelling on the left maxillary region revealed mesenchymal proliferation with evidence of bone remodeling, epithelial cell proliferation, and mild macrophagic inflammation. The possibility of a well-differentiated salivary gland carcinoma could not be ruled out. While osteoblast-like cells could have represented a neoplastic population, they exhibited only mild criteria of malignancy, as observed with areas of remodeled bone. The possibility that the mesenchymal cell population represented neoplastic cells (e.g., fibrosarcoma) could not be ruled out. Regional lymph node cytology was not performed. The fungal culture and Brucella testing were both negative. The Canis Brucella Multiplex Assay is a Brucella Agar Gel Immunodiffusion test that screens for antibodies detected against two recombinant antigens derived from *B. canis*. A negative test result for both antibodies provides a strong indication that antibodies against Brucella were not present in the sample.

**Figure 3:**
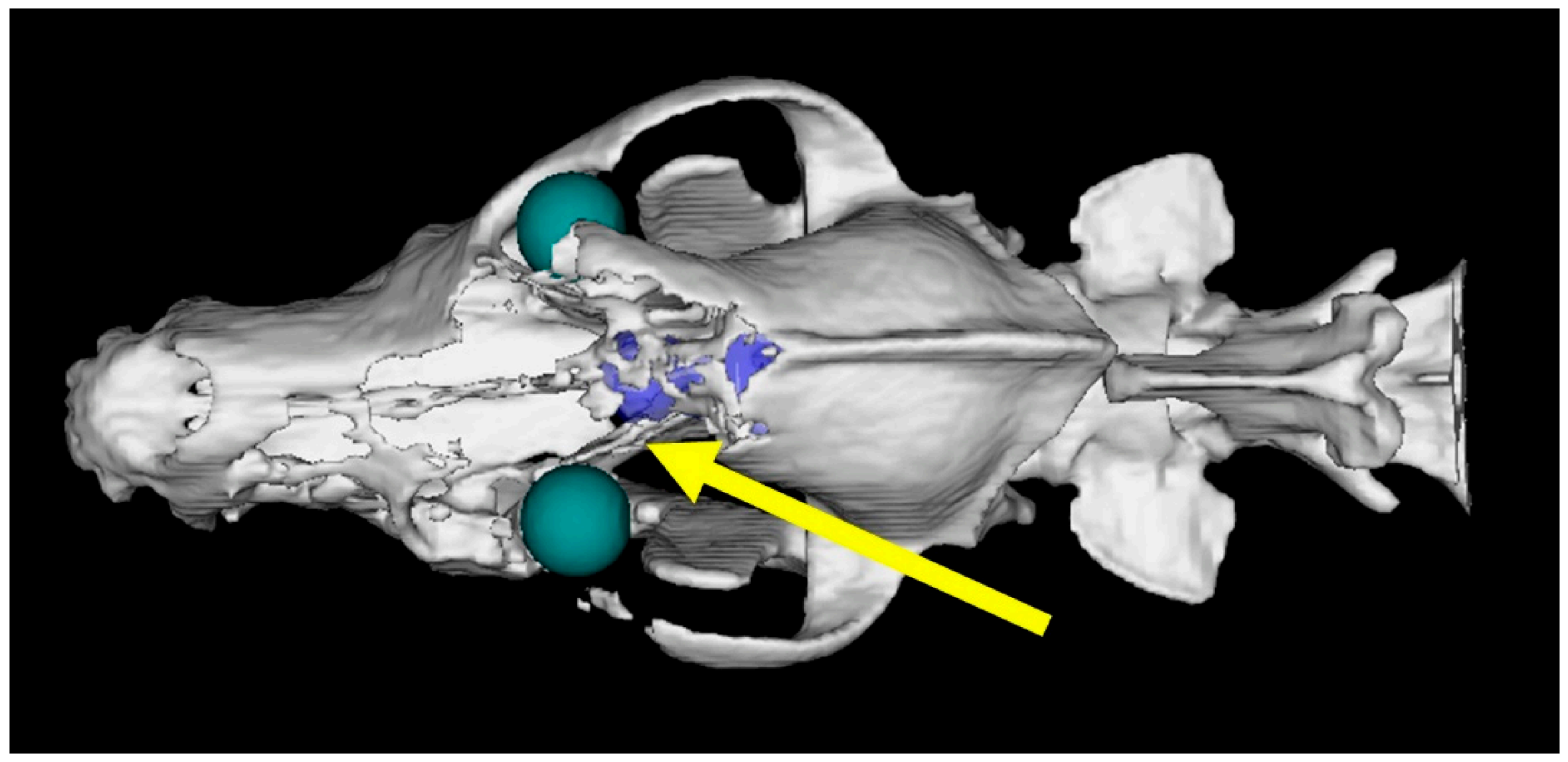
Dorsal-view, three-dimensional image using radiation therapy planning software (Eclipse software, Varian Medical Systems, version 16.0). The arrow represents one sampling site caudal to the left eye, where there was bony destruction of the calvarium. The regions in blue/purple represent exposed brain tissue due to extensive calvarial lysis of bone (white).

**Figure 4:**
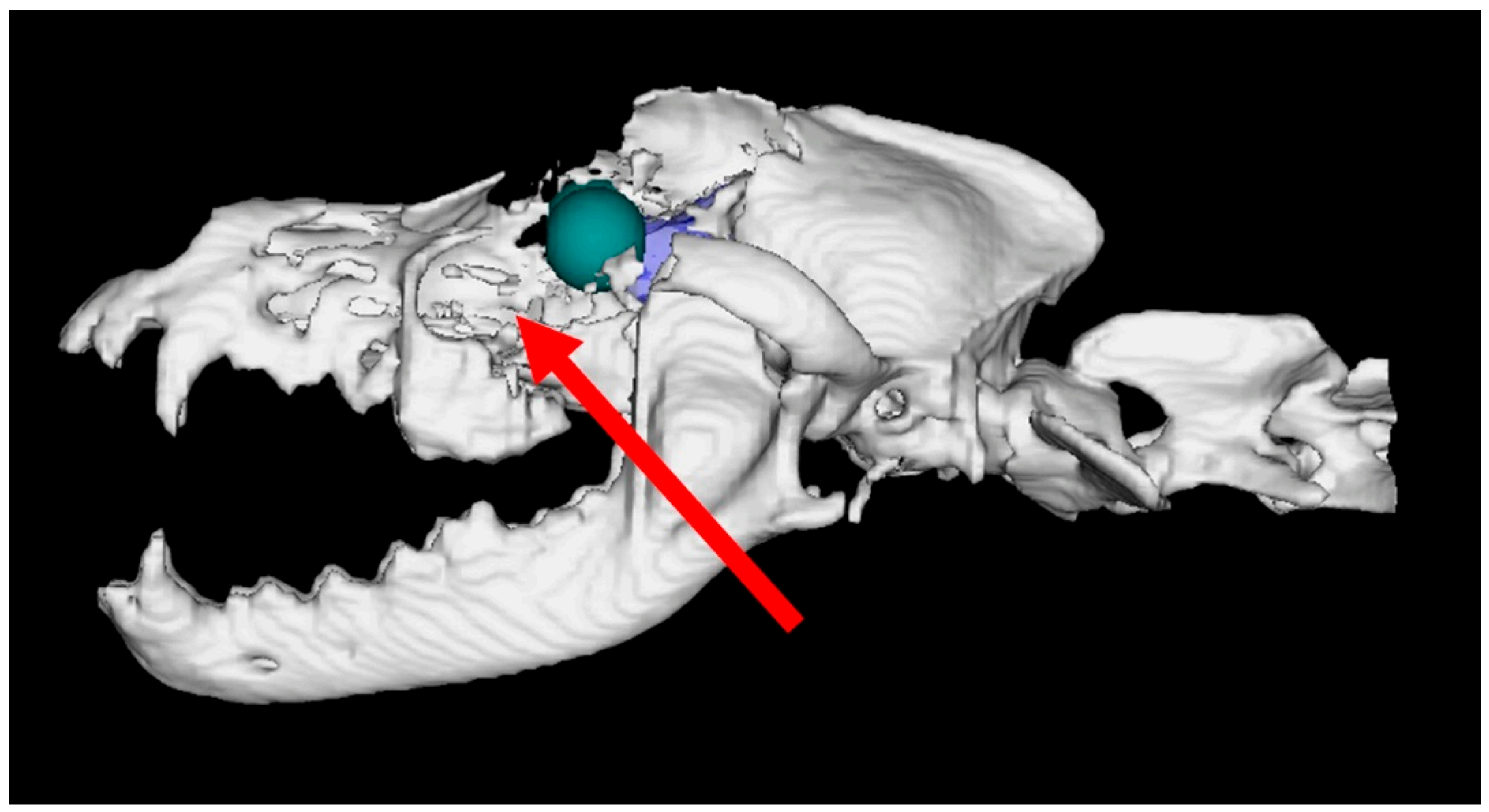
Sagittal, three-dimensional reconstruction that shows where the second sample was taken rostral to the left eye (red arrow).

Surgical biopsy was recommended because ultrasound-guided cytology was inconclusive at the time of CT. An incision was made on the dorsal midline over the affected tissue caudal to the left eye, where the calvarium was palpated abnormally (Figure 3). After being fixed in formalin, six samples were measured: two bony specimens measured 1.0 × 1.0 × 0.5 cm and 1.5 × 1.5 × 0.5 cm; four were soft and ranged from 1.0 to 1.5 cm. A second incision and biopsy were made rostral to the left eye over the area of focal facial swelling (Figure 4). A total of nine samples were biopsied: four soft tan and brown pieces measured 0.5 to 1.0 cm, and five boney tan and brown tissues measured 0.5 to 1.5 cm after formalin fixation. Muscle and skin were retracted, allowing the abnormal bone to be visualized for biopsy collection. Biopsy was performed using a #10 blade and Rongeur bone forceps for both sites. Biopsies were routinely fixed in 10% neutral buffered formalin for approximately 24 h, followed by processing. Routine processing included a 12 h cycle of progressive rehydration in a series of graded ethanol, clearing with xylene, and infiltration with paraffin wax.

**Figure 5:**
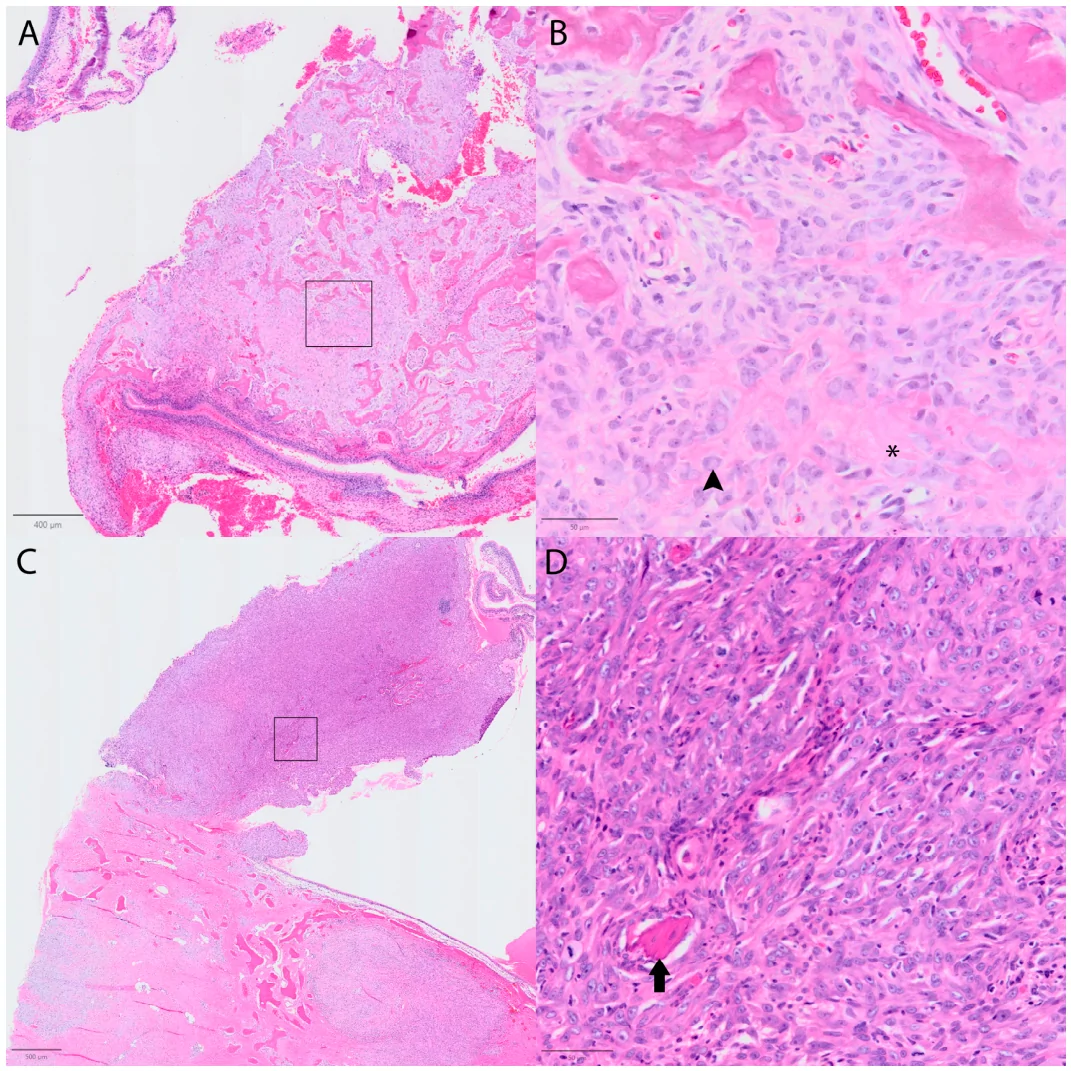
(**A**). The neoplasm produces anastomosing trabeculae of osteoid. (**B**). Higher magnification of box in A. The osteoid matrix (*), located between neoplastic cells with osteoblastic differentiation (arrowhead), becomes mineralized (top). (**C**). In most areas, the neoplasm fails to produce osteoid. (**D**). Higher magnification of box in C. Neoplastic cells in areas lacking osteoid production with an entrapped fragment of non-neoplastic bone (arrow).

Following processing, biopsy tissue was embedded in paraffin. Formalin-fixed paraffin-embedded sections were cut at 5 µm, mounted on positively charged glass slides, and stained with hematoxylin and eosin for histological examination. In total, thirteen sections from the first sample (Figure 3) and twelve sections from the second sample (Figure 4) were examined histologically. Histologically, the left facial mass was a non-encapsulated, poorly demarcated, densely cellular neoplasm expanding the submucosal connective tissue beneath the respiratory epithelium. The neoplasm was composed of plump spindle cells arranged in haphazard, disorganized streams within a collagenous stroma, separated by entrapping anastomosing mineralizing trabeculae and wispy streams of brightly eosinophilic, homogeneous to fibrillar osteoid matrix (Figure 5A,B). Most sections of the neoplasm lacked osteoid matrix production (Figure 5C,D). Neoplastic cells had a scant amount of eosinophilic cytoplasm with poorly defined cell borders. Nuclei were ovoid to elongate with finely stippled chromatin with 1–2 basophilic nucleoli, with a perinuclear clear zone consistent with a Golgi zone. Anisocytosis and anisokaryosis are marked. There were 15 mitotic figures in ten FN22 high-power fields (2.37 mm^2^) in the area of highest mitotic activity. Sections from the nasal region were consistent with a histologically similar neoplasm and granulation tissue. Histologic findings were most consistent with a variably productive osteoblastic OSA. A soft tissue sarcoma was considered a much less likely differential diagnosis given the CT imaging being unable to detect a soft tissue mass effect.

Treatment options were discussed with the owner, which included local therapy (surgery or radiation therapy) and systemic therapy (chemotherapy and bisphosphonates). Our institution’s soft tissue surgeons determined this case was not surgical, given the pre-existing extensive bone lysis and that resection was unlikely to result in complete resection. Similarly for radiation therapy, there were great concerns for risk of side effects to the surrounding normal tissues, as well as difficulty evaluating the future treatment efficacy because radiation side effects to bone often result in further lysis. The owner had also expressed concerns in managing or identifying potential side effects of radiation. Therefore, radiation therapy was not pursued. Given that the benefit of chemotherapy as a sole form of treatment for axial osteosarcomas is not established, this systemic treatment was not pursued. Lastly, zoledronate (a bisphosphonate) infusions were presented as a low risk of side effect approach with a potential benefit in delaying further bone loss.

The first intravenous zoledronate at 0.1 mg/kg diluted in 100 mL of 0.9% NaCl given over 15 min was administered by the Radiation Oncology service at 32 days post-diagnosis, and the point-of-care bloodwork was unremarkable. The dog returned on 67 days post-diagnosis for the second course of zoledronate, along with point-of-care blood analysis. This showed a metabolic alkalosis (pH 7.476, pO2 98.3, pCO2 25.8 and cSO2 98.2), but bloodwork was otherwise unremarkable. At 81 days following diagnosis, the client reported that the dog was doing well, eating and drinking normally, and maintaining normal energy levels. The dog received the third zoledronate infusion 102 days post-diagnosis by the Radiation Oncology service. Recheck point-of-care blood analysis during that visit was completely unremarkable, and physical examination findings remained static. At that time, the dog was still on carprofen at 1.6 mg/kg by mouth twice daily, gabapentin at 19 mg/kg by mouth twice daily, fluoxetine at 1.3 mg/kg by mouth once daily and topical Ophytrium 0.5% and chlorhexidine 3% wipes as needed for skin irritation that was historical.

At 132 days post-diagnosis, the dog presented to Emergency Service for acute vestibular signs and inability to stand. Physical examination findings on presentation included horizontal nystagmus and an increased rectal temperature at 102.8 °F (39.3 °C). Due to concerns about quality of life, the owners elected to humanely euthanize the dog during this visit. No necropsy was performed. The survival time calculated from the date of CT imaging was 159 days, or 132 days from the date of biopsy.

## 3. Discussion

The patient described here had craniomaxillofacial OSA with no radiographic evidence of metastasis at the time of diagnosis. This presentation of OSA tends to be locally aggressive with a high chance of recurrence after attempted excision. Treatment is typically guided towards surgical excision, as local disease is most commonly the cause of death. Other treatments, such as chemotherapy and stereotactic body radiation therapy, either in combination or solely, have been described for craniomaxillofacial OSA but have not been shown to yield better outcomes, given the median survival time reported was 232 days, with 63% of dogs still experiencing local disease progression as the cause of death [10]. At the time of presentation to the Radiation Oncology service, the dog was given a zoledronate infusion, which is a third-generation bisphosphonate to slow the breakdown of bone, to impart anti-tumor effects and promote osteoclast apoptosis. Bisphosphonates are synthetic analogs of pyrophosphates that inhibit osteoclast function. The main goal of bisphosphonate use in dogs and humans with OSA is analgesia due to its antiresorptive mechanism of action [11]. While there are reports of other bisphosphonates used in veterinary medicine, zoledronate has the benefit of less infusion time in hospital (15 min) compared to pamidronate (infusion over 2 h). This allows patients the ability to spend as little time in hospital as possible.

Non-stereotactic hypofractionated radiation therapy (RT) was discussed with the owner for local control, but concerns were expressed regarding the primary lytic presentation of this patient’s OSA and the risks of radiation side effects given the extensive tumor involvement. The primary lytic nature of this patient’s bone cancer would also make therapeutic monitoring via CT scan very challenging, since concurrent bacterial/fungal osteomyelitis (secondary to an irradiated nasal mucosal epithelium) or side effects of osteoradionecrosis or both cannot be differentiated [12]. In addition, tumor progression or osteoradionecrosis would not be differentiated on CT or positron emission tomography imaging, given the primary nature of this cancer’s bone lysis pattern [13]. Based on analysis of CT scan images, the most significant organs at risk of radiation side effects for this patient were the left eye, left optic nerve, other facial nerves (cranial nerves VII and V), brain (olfactory bulb and frontal lobe), bones (frontal bone, orbital part of the frontal bone, orbital plate of the ethmoid bone, frontal process of the maxilla, palatine process of the maxilla, the zygomatic bone, and the cribriform plate), oral mucosa, skin, and nasal cavity. The severity and types of side effects depend on the dose of radiation given and the organ type [14]. Given the risk of side effects and lack of imaging features for monitoring tumor response to radiation, it was not elected to pursue this treatment modality.

An elevation in ALP at presentation to the Internal Medicine department was documented. ALP is an omnipresent enzyme found primarily in the intestine, kidney, liver, and bone [15]. Serum total ALP activity is a practical prognostic factor because it is readily quantifiable as a component of a routine chemistry panel at many veterinary diagnostic laboratories. One study evaluated the prognostic significance of serum total ALP and each of its constituent isoenzymes in dogs with appendicular OSA treated with amputation and cisplatin/doxorubicin chemotherapy. Results showed that the median survival time for the 61 dogs was 10.7 months, and 45% of dogs were alive one-year after amputation [15]. However, serum ALP concentration has not been consistently identified as a prognostic factor in canine or human OSA studies [16]. Although serial ALP evaluations were not done with every visit, this case’s initial elevation of ALP around the time of diagnosis could have been a contributing clue to the diagnosis of OSA.

Finally, the vestibular signs that the dog exhibited prior to being euthanized were likely due to encroachment of the lytic lesions on his skull into the brain; however, necropsy was not performed. The dog exhibited peripheral and central vestibular signs of ataxia and nystagmus, respectively; therefore, other possible causes of the acute vestibular signs include otitis media or otitis interna, as the dog had a chronic history of ear and skin infections, idiopathic or potentially indicative of a vascular event (i.e., stroke). There was no inciting event prior to his vestibular signs; therefore, a trauma etiology is less likely to have caused these signs, though it cannot be ruled out due to the lack of bone surrounding the brain from neoplastic osteolysis.

## 4. Conclusions

This case report illustrates the unusual presentation (primarily lytic bone) of an osteoblastic osteosarcoma of a dog’s skull. With medical management of bisphosphonate treatments alone, the short survival time of 132 days from the date of biopsy, or 159 days calculated from the date of CT imaging for this case report was similar to the median survival time reported in dogs with axial skeletal OSA that received surgery or radiation therapy, although this comparison cannot be taken as a generalized outcome for all axial osteosarcomas, given our study only involves a single individual.

## Data Availability

The original contributions presented in this study are included in the article. Further inquiries can be directed to the corresponding author(s).

## Abbreviations

The following abbreviations are used in this manuscript:

CT	Computed Tomography
OSA	Osteosarcoma
ALP	Alkaline Phosphatase
RT	Radiation Therapy

## References

[1] Ehrhart N.P., Christensen N.I., Fan T.M., Vail D.M., Thamm D.H., Liptak J.M. (2020). Tumors of the skeletal system. Withrow & MacEwen’s Small Animal Clinical Oncology.

[2] Spodnick G., Berg J., Rand W.M., Schelling S.H., Couto G., Harvey H.J., Henderson R.A., MacEwen G., Mauldin N., McCaw D.L. (1992). Prognosis for dogs with appendicular osteosarcoma treated by amputation alone: 162 cases (1978–1988). J. Am. Vet. Med. Assoc..

[3] Heyman S.J., Diefenderfer D.L., Goldschmidt M.H., Newton C.D. (1992). Canine axial skeletal osteosarcoma. A retrospective study of 116 cases (1986 to 1989). Vet. Surg..

[4] Coyle V.J., Rassnick K.M., Borst L.B., Rodriguez C.O., Northrup N.C., Fan T.M., Garrett L.D. (2015). Biological behaviour of canine mandibular osteosarcoma. A retrospective study of 50 cases (1999–2007). Vet. Comp. Oncol..

[5] Dickerson M.E., Page R.L., Ladue T.A., Hauck M.L., Thrall D.E., Stebbins M.E., Price G.S. (2001). Retrospective analysis of axial skeleton osteosarcoma in 22 large-breed dogs. J. Vet. Intern. Med..

[6] Selmic L.E., Lafferty M.H., Kamstock D.A., Garner A., Ehrhart N.P., Worley D.R., Withrow S.J., Lana S.E. (2014). Outcome and prognostic factors for osteosarcoma of the maxilla, mandible, or calvarium in dogs: 183 cases (1986–2012). J. Am. Vet. Med. Assoc..

[7] Wallace J., Matthiesen D.T., Patnaik A.K. (1992). Hemimaxillectomy for the treatment of oral tumors in 69 dogs. Vet. Surg..

[8] Kosovsky J.K., Matthiesen D.T., Patnaik A.K. (1991). Results of partial mandibulectomy for the treatment of oral tumors in 142 dogs. Vet. Surg..

[9] Straw R.C., Powers B.E., Klausner J., Henderson R., Morrison W., McCaw D., Harvey H., Jacobs R., Berg R. (1996). Canine mandibular osteosarcoma: 51 cases (1980–1992). J. Am. Anim. Hosp. Assoc..

[10] Altwal J., Lee B.I., Boss M.K., LaRue S.M., Martin T.W. (2024). Outcomes of 35 dogs with craniomaxillofacial osteosarcoma treated with stereotactic body radiation therapy. Vet. Comp. Oncol..

[11] Fan T.M., de Lorimier L.P., Garrett L.D., Lacoste H. (2008). The bone biologic effects of zoledronate in healthy dogs and dogs with malignant osteolysis. J. Vet. Intern. Med..

[12] Gieger T.L., Haney S.M., Nolan M.W. (2022). Re-irradiation of canine non-lymphomatous nasal tumours using stereotactic radiation therapy (10 Gy x 3) for both courses: Assessment of outcome and toxicity in 11 dogs. Vet. Comp. Oncol..

[13] Alhilali L., Reynolds A.R., Fakhran S. (2014). Osteoradionecrosis after radiation therapy for head and neck cancer: Differentiation from recurrent disease with CT and PET/CT imaging. Am. J. Neuroradiol..

[14] Poirier V.J., Keyerleber M., Gordon I.K., Turek M.M., Kent M.S., Bentley E., Lawrence J. (2023). ACVR and ECVDI consensus statement: Reporting elements for toxicity criteria of the veterinary radiation therapy oncology group v2.0. Vet. Radiol. Ultrasound.

[15] Garzotto C.K., Berg J., Hoffmann W.E., Rand W.M. (2000). Prognostic significance of serum alkaline phosphatase activity in canine appendicular osteosarcoma. J. Vet. Intern. Med..

[16] Holmes K.E., Thompson V., Piskun C.M., Kohnken R.A., Huelsmeyer M.K., Fan T.M., Stein T.J. (2015). Canine osteosarcoma cell lines from patients with differing serum alkaline phosphatase concentrations display no behavioural differences in vitro. Vet. Comp. Oncol..

